# Immunometabolism characteristics and a potential prognostic risk model associated with TP53 mutations in breast cancer

**DOI:** 10.3389/fimmu.2022.946468

**Published:** 2022-07-22

**Authors:** Mengping Jiang, Xiangyan Wu, Shengnan Bao, Xi Wang, Fei Qu, Qian Liu, Xiang Huang, Wei Li, Jinhai Tang, Yongmei Yin

**Affiliations:** ^1^ Department of Oncology, The First Affiliated Hospital of Nanjing Medical University, Nanjing, China; ^2^ The First Clinical College of Nanjing Medical University, Nanjing, China; ^3^ School of Electro-mechanical Engineering, Guangdong University of Technology, Guangzhou, China; ^4^ Department of General Surgery, The First Affiliated Hospital of Nanjing Medical University, Nanjing, China; ^5^ Jiangsu Key Lab of Cancer Biomarkers, Prevention and Treatment, Collaborative Innovation Center for Personalized Cancer Medicine, Nanjing Medical University, Nanjing, China

**Keywords:** TP53, prognostic model, immune heterogeneity, metabolic heterogeneity, breast cancer

## Abstract

*TP53*, a gene with high-frequency mutations, plays an important role in breast cancer (BC) development through metabolic regulation, but the relationship between TP53 mutation and metabolism in BC remains to be explored. Our study included 1,066 BC samples from The Cancer Genome Atlas (TCGA) database, 415 BC cases from the Gene Expression Omnibus (GEO) database, and two immunotherapy cohorts. We identified 92 metabolic genes associated with TP53 mutations by differential expression analysis between TP53 mutant and wild-type groups. Univariate Cox analysis was performed to evaluate the prognostic effects of 24 TP53 mutation-related metabolic genes. By unsupervised clustering and other bioinformatics methods, the survival differences and immunometabolism characteristics of the distinct clusters were illustrated. In a training set from TCGA cohort, we employed the least absolute shrinkage and selection operator (LASSO) regression method to construct a metabolic gene prognostic model associated with TP53 mutations, and the GEO cohort served as an external validation set. Based on bioinformatics, the connections between risk score and survival prognosis, tumor microenvironment (TME), immunotherapy response, metabolic activity, clinical characteristics, and gene characteristics were further analyzed. It is imperative to note that our model is a powerful and robust prognosis factor in comparison to other traditional clinical features and also has high accuracy and clinical usefulness validated by receiver operating characteristic (ROC) and decision curve analysis (DCA). Our findings deepen our understanding of the immune and metabolic characteristics underlying the TP53 mutant metabolic gene profile in BC, laying a foundation for the exploration of potential therapies targeting metabolic pathways. In addition, our model has promising predictive value in the prognosis of BC.

## Introduction

Breast cancer (BC) is the most common cancer that threatens women’s health around the world (1). Thanks to the spread of early diagnosis based on advanced medical technology and comprehensive and precise treatment, the survival of early BC patients has greatly improved (2, 3). However, a certain number of BC patients relapse following therapy or develop metastatic cancer within a short period, with unsatisfactory treatment efficacy and only a 30% 5-year survival rate, which has become a clinical challenge nowadays (4). Several lines of evidence have shown that BC progression is associated with changes in cellular metabolism, and a high level of metabolic heterogeneity within breast tumors is one of the main causes of these changes (5–8). Over the past decades, burgeoning studies have focused on the metabolic heterogeneity of BC, due to its tumor-promoting function and important impact on prognosis and therapeutic response, and attempted to overcome it to develop novel approaches, including genomic profiling, the development of biomarkers, and targeted metabolic therapies (9, 10). Nevertheless, there are few types of research on the detailed elaboration of the association between metabolic phenotypes and BC prognosis.

*TP53* (P53) gene, widely regarded as a tumor suppressor gene, is the most frequently mutated gene in cancer (11). Protein p53 encoded by human gene *TP53* has diverse biological functions, primarily acting as a transcription factor to initiate gene transcription and participate in cell cycle arrest, metabolism, DNA repair, cell senescence, apoptosis, and ferroptosis (12, 13). The majority of TP53 mutations in human cancer are missense mutations in its DNA-binding domain that generate mutant p53 protein, thus affecting its transcriptional ability and abnormal downstream signaling pathway (14). Numerous studies have revealed that mutant p53 can lose their tumor-suppressive function and obtain dominant−negative activities that are independent of wild−type p53, which may confer them oncogenic functions to participate in cancer development (15, 16). Although the clinical relevance of TP53 mutation status in BC has been recently debated, it is also worth noting that TP53 mutations are linked with worse survival in BC, supported by credible results from several large samples of clinical data (17–19). Therefore, more efforts to further discover the roles of TP53 in BC progression are warranted.

As observed in multiple studies, mutant p53 may exert its oncogenic functions primarily by regulating cancer metabolism (20, 21). Thus, we speculate that the prognosis of BC patients with TP53 mutations may be related to cancer metabolism regulation mediated by mutant p53. In our research, a comprehensive analysis of TP53 gene status in BC was conducted to reveal the relationship between TP53 mutations and metabolic phenotypes. Furthermore, a TP53-related metabolic gene profile containing 24 metabolic genes was identified and characterized by a high degree of metabolic and immune microenvironmental heterogeneity according to differential gene expression analysis in patients with TP53 mutations and TP53 wild type. Importantly, we developed a prognostic risk score model based on TP53-associated metabolic gene profiles that could contribute to risk stratification in BC patients and guidance of clinical decision making. The validation of The Cancer Genome Atlas (TCGA) and Gene Expression Omnibus (GEO) data proves the excellent predictive value of this model for BC prognosis.

## Materials and methods

### Sources of breast cancer datasets and preprocessing

The RNA sequencing data (HTSeq-Counts) and copy number variation (CNV) data of BC were retrieved from TCGA website (https://portal.gdc.cancer.gov/) and the University of California, Santa Cruz (UCSC) Xena website, respectively. In addition, Breast Invasive Carcinoma TCGA PanCancer data of 1,084 samples containing somatic mutation data, especially *TP53* gene condition, and the matched clinical information were downloaded from cBio Cancer Genomics Portal (http://cbioportal.org/), which collected a large number of comprehensive multidimensional cancer genome databases (22, 23). Patients without survival data were removed from further analysis. To facilitate comparability among samples, HTSeq-Counts data were normalized to transcripts per kilobase million (TPM) values and transformed to log2TPM for the following analysis (24).

For microarray data from the GEO database (https://www.ncbi.nlm.nih.gov/geo/), we downloaded GSE20685 (n = 327) and GSE20711 (n = 90) datasets with the matrix files of the gene expression profile and full clinical information based on the same platform GPL570 and developed a GEO cohort of 415 BC cases. Gene expression data of the GEO cohort were log2-transformed. Batch effects from non-biological technical biases were corrected by using the “ComBat” algorithm of the “SVA” package.

To validate the ability of our model to predict response to anti-PD-1/L1 therapy, we adopted two immunotherapeutic cohorts after a series of searches: IMvigor210 cohort (advanced urothelial cancer treated with atezolizumab) (25) and GSE78220 (metastatic melanoma with the intervention of pembrolizumab (26). The count data of IMvigor210 and microarray data of GSE78220 were converted to log2TPM and log2-transformed as described above, respectively.

Generally, the average expression value of genes was used in the present study if gene duplication was detected. Data used in this study are publicly available from TCGA and GEO databases.

### Gene set variation analysis

We performed gene set variation analysis (GSVA) enrichment analysis among different groups or patterns to reveal metabolic heterogeneity in BC by the “GSVA” R packages, as visualized in the heatmap (27). The file of “c2.cp.kegg.v7.4.symbols” was obtained from the MSigDB database for GSVA. We screened for statistically significant pathways between different clusters depending on the adjusted p < 0.05.

### Estimation of tumor microenvironment cell infiltration

To describe meaningful variations among distinct groups or clusters in tumor microenvironment (TME) cell infiltration, a single-sample gene set enrichment analysis (ssGSEA) algorithm was employed to quantify the relative abundance of 28 subpopulations of tumor-infiltrating lymphocytes (TILs) in the BC TME for differential analysis. The natural killer T cells, activated CD8+ T cells, activated CD4+ T cells, activated dendritic cells, macrophages, and other representative human immune cell subtypes are included in this study (28, 29).

### Differentially expressed genes associated with TP53 condition

We classified 1,003 BC patients with gene expression data and TP53 mutation information from TCGA cohort into TP53 wild-type (n = 659) and TP53 mutant (n = 344) groups. The “edgeR” package was conducted on the real count data of BC samples to identify differentially expressed genes (DEGs) between the two groups in BC (|logFC|>1 and adjusted p < 0.05). The results were visualized in volcano plots and heatmap.

### Metabolic gene source and functional analysis

With the use of the Kyoto Encyclopedia of Genes and Genomes (KEGG) metabolic pathway-related gene sets on the KEGG website (https://www.kegg.jp/), a total of 3,067 metabolic genes were extracted from 86 KEGG metabolic pathways and collected (**Supplementary Table 1**) (30). The intersection of the DEGs and metabolic genes were selected as metabolism-related genes (MRGs) associated with the TP53 condition for subsequent analysis. By using the “clusterProfiler” R package, the analysis of KEGG pathways was utilized to explore the significant pathways associated with TP53-related MRGs, highlighting the biological implications of the prognostic model (31). The notable pathways were shown in the bar plot using “ggplot2” R packages.

### Consensus clustering

Consensus clustering was performed to identify distinct TP53-related metabolic patterns based on the expression of 24 TP53-associated MRGs by the k-means method. Notably, the optimal cluster number of clusters depended on the consensus clustering algorithm by applying the “ConsensuClusterPlus” package. We carried out 1,000 repetitions to further verify the robustness of our classification (32).

### Gene set enrichment analysis

Based on the gene set database of “c2.cp.kegg.v7.5.1.symbols,” GSEA version 4.2.2 (33) was used to discover different underlying mechanisms of enrichment in the high- and low-risk groups, with a particular focus on MRG sets. In terms of key parameter setting, “high risk versus low risk” was assigned the phenotypic label, and the number of random sample permutations was set at 1,000.

### Development of the risk score model based on TP53-related metabolism-related genes

We selected a total of 1,039 BC cases with gene expression data and survival information from TCGA database as the training cohort and 415 BC patients from the GEO cohort as the testing cohort. Before subsequent analysis, the gene expression data of two cohorts were corrected by the “scale” method. In the training cohort, we performed the prognostic analysis for TP53-related MRGs with an application of the univariate Cox regression model. Those TP53-related MRGs with p-values <0.05 were regarded as significant in statistics and then enrolled into the least absolute shrinkage and selection operator (LASSO) regression analysis for the foundation of the prognostic risk score model.


$$Risk\;Score=\sum_{i=1}^{n}KiXi$$

where n, Ki, and Xi represent the number of included genes, the coefficient index, and the gene expression, respectively. With median risk score as a cutoff point, the patients in the training cohort were stratified into low- and high-risk subgroups. We attempted to evaluate the predictive accuracy of the model *via* time receiver operating characteristic (ROC) analysis.

### Statistical analysis

All statistical tests and drawings were conducted using R statistical software (version 4.1.2). Two-group comparisons were carried out using Student’s t-test and Wilcoxon’s test, and the Kruskal–Wallis tests for comparisons of three or more groups. The overall survival (OS) differences among diverse groups were analyzed by the Kaplan–Meier method, and the statistical significance of differences was identified using the log-rank test. Univariate Cox regression analysis was performed to assess the prognostic value of genes and visualized with forest plots by “Survival” R packages. The measure of the correlation between 24 metabolic genes was implemented using “psych” R packages. The waterfall plots for the mutation landscape of patients with or without TP53 mutations in TCGA cohort were generated *via* the function of the “maftools” R package. Univariate and multivariate Cox regression analyses were executed to assess whether the risk score in combination with clinical characteristics had independent prognostic power. A p < 0.05 was considered statistically significant.

## Results

### Functional changes associated with TP53 mutations in breast cancer

**Figure 1:**
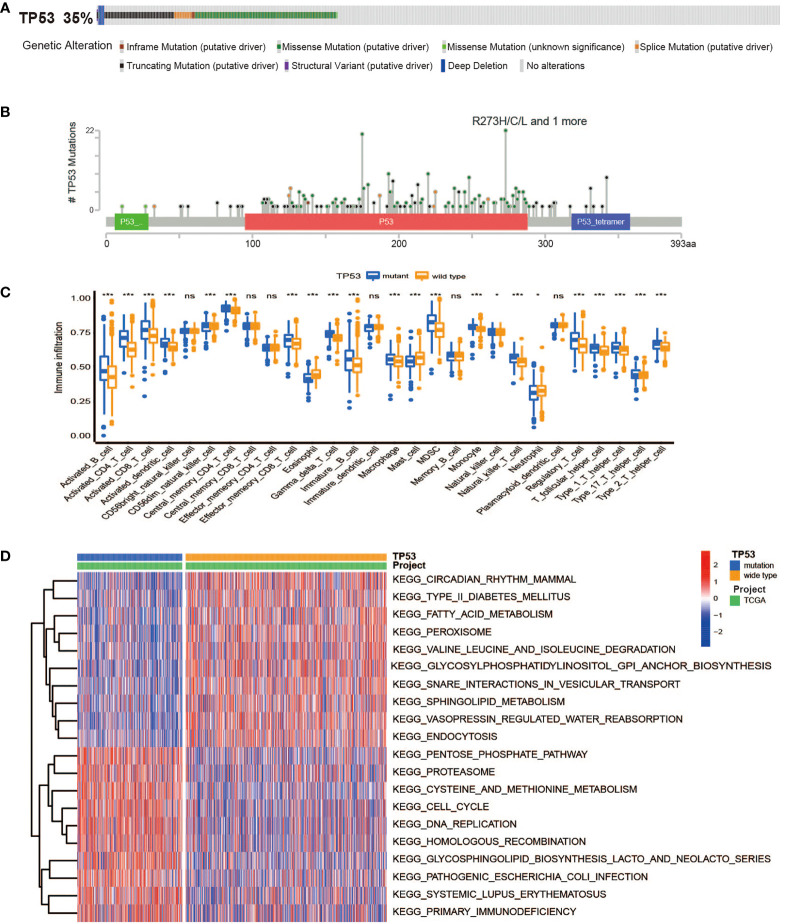
The overall characteristics of TP53 mutations in breast cancer. **(A)** Frequency and types of *TP53* gene alterations in TCGA Breast Invasive Carcinoma samples from cBioPortal. **(B)** The distribution of amino acids in TP53 protein in TCGA Breast Invasive Carcinoma samples from cBioPortal. **(C)** The abundance of each TME infiltrating cell between TP53 mutant and wild-type groups (ns, no significance; *p < 0.05; ***p < 0.001). **(D)** Heatmap for GSVA enrichment analysis showing the activation states of biological pathways between TP53 mutant and wild-type groups. TCGA, The Cancer Genome Atlas; TME, tumor microenvironment; GSVA, gene set variation analysis.

In TCGA cohort, TP53 mutation frequency was up to 35%, among which missense mutations accounted for the largest proportion, as shown in **Figure 1A**. Analysis of p53 protein mutation sites displayed the most mutation sites in the DNA-binding domain that initiated their principal functions, indicating that the functional changes of mutant p53 were affected by DNA-binding ability (**Figure 1B**). In our research, no remarkable variable in OS for BC patients was observed between the TP53 mutated and unmutated groups (log-rank p > 0.05, **Supplementary Figure 1**). To identify differences in the biological pathway activities of the two groups, we employed GSVA to determine that BC cases with TP53 mutation exhibit enrichment of energy metabolism (galactose metabolism, and pentose phosphate pathway), amino acid metabolism (methionine and cysteine), and immunologic function (natural killer cell-mediated cytotoxicity), as compared to those without TP53 mutation (**Figure 1D**, **Supplementary Table 2**). In addition, we found a high degree of immune cell infiltration in the TP53 mutation group by ssGSEA (**Figure 1C**). In brief, our results confirmed that TP53 mutations led to changes in metabolic and immune characteristics in BC, which may be biological hallmarks of malignancy.

### The identification of metabolism-related differentially expressed genes based on TP53 status

**Figure 2:**
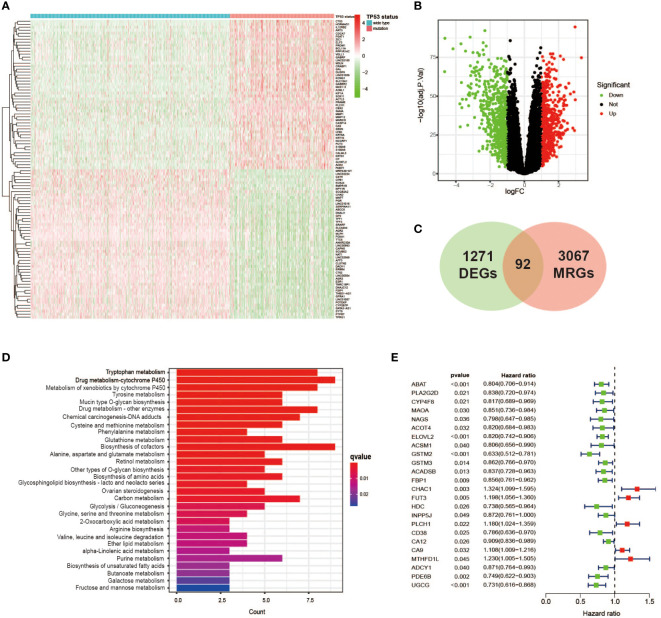
Identification of TP53 mutation-related metabolic gene profile. **(A)** Heatmap of the DEGs between TP53 mutant and wild-type groups in breast cancer cases. **(B)** A volcano plot exhibiting the identified DEGs including upregulated (red) and downregulated (green) genes. **(C)** Venn diagram summarizes the TP53-related metabolic genes intersected by 3,067 MRGs and 1,271 DEGs. **(D)** KEGG analysis of 92 metabolic genes related to TP53 showing the main significantly enriched pathways. **(E)** Overall survival in univariate Cox regression of TP53-related metabolic genes. DEGs, differentially expressed genes; MRGs, metabolism-related genes; KEGG, Kyoto Encyclopedia of Genes and Genomes.

DEG analysis was performed between the TP53 mutant group and TP53 wild-type group using the “edgeR” package among a total of 1,003 samples with complete data on gene expression and TP53 status, and we finally identified 1,271 DEGs related to the TP53 condition (false discovery rate (FDR) < 0.05 and |log2 FC| > 1.0, **Figures 2A**, **B**). To further analyze the relationship between TP53 mutations and metabolism in BC, 3,067 MRGs assigned to metabolic pathways were obtained from the official website of the KEGG database, and then 92 TP53-related DEGs relevant to metabolic regulation were ultimately ascertained (**Figure 2C**). Among them, 47 genes were upregulated (FDR < 0.05 and log2 FC > 1.0), and 45 genes were downregulated (FDR < 0.05 and log2 FC < 1.0). In addition, KEGG pathway analysis revealed that 92 metabolism-related DEGs (MRDEGs) were mainly involved in 20 metabolic pathways, including amino acid metabolism (tryptophan, tyrosine, cysteine, methionine, glutathione, glutamate, serine, threonine, and arginine metabolism), glucose metabolism (glycolysis, gluconeogenesis, galactose, fructose, and mannose metabolism), and tricarboxylic acid (TCA) cycle and followed by oxidative phosphorylation, lipid metabolism (biosynthesis of unsaturated fatty acids), and drug metabolism (**Figure 2D**, **Supplementary Table 3**). Similar to those of other studies, these results demonstrated the pivotal role of TP53 status in metabolic regulation for BC patients.

### The features of metabolic genes profile with TP53 mutations

**Figure 3:**
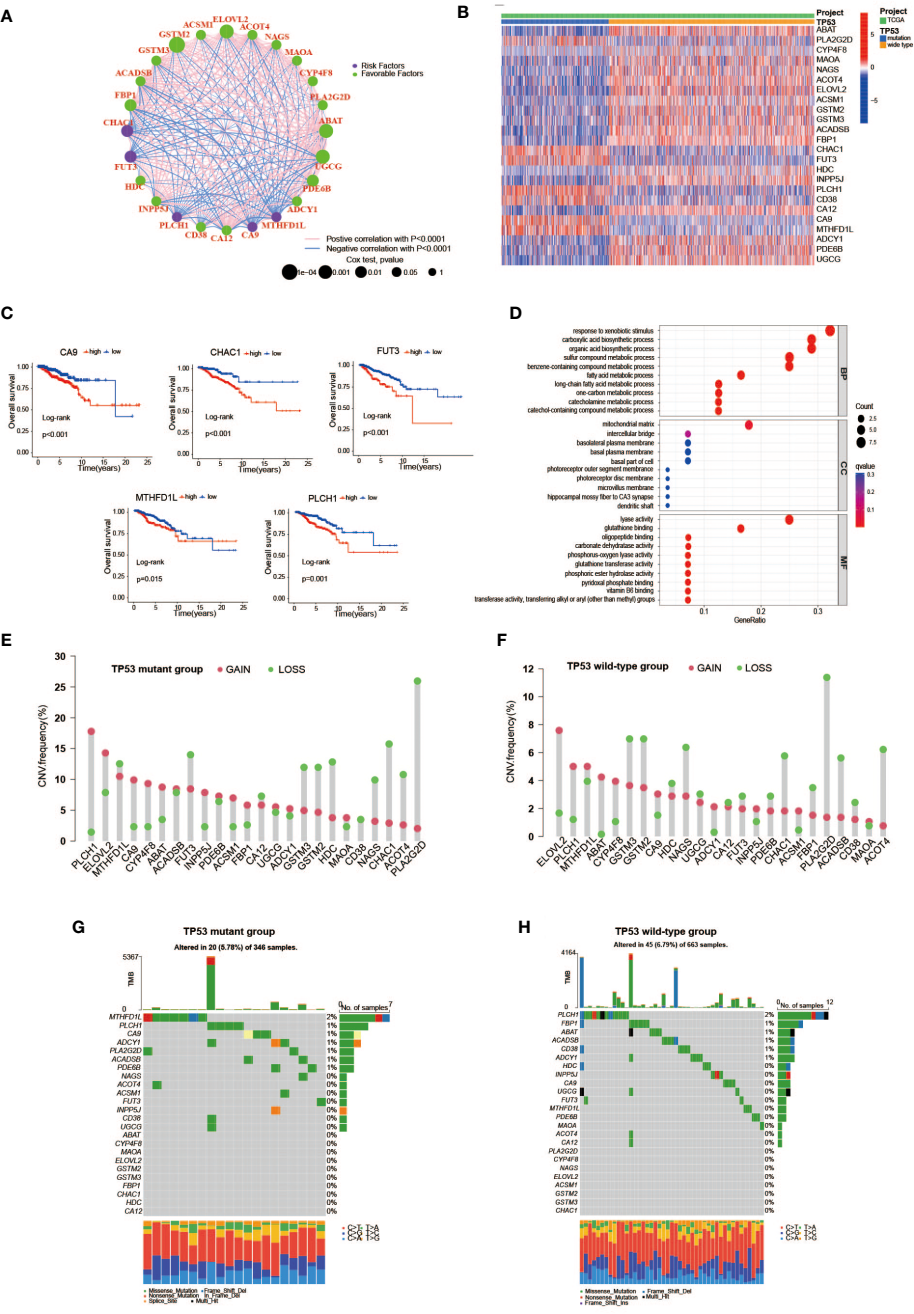
The features of TP53 mutation-related metabolic gene profile. **(A)** A network of the interactions among the 24 metabolic genes associated with TP53 mutations. **(B)** Heatmap of TP53 mutation-related metabolic gene expression profile between TP53 mutant and wild-type groups. Seven upregulated genes including *CA9*, *PLCH1*, *FUT3*, *CHAC1*, *CD38*, *MTHFD1L*, and *PLA2G2D* in the TP53 mutant group. **(C)** Kaplan–Meier survival analysis of breast cancer patients with high and low gene expression of 5 prognostic risk genes (*CA9*, *CHAC1*, *FUT3*, *MTHFD1L*, and *PLCH1*). **(D)** GO enrichment analysis of TP53 mutation-related metabolic gene profile in cellular component terms, biological process terms, and molecular function terms. **(E, F)** Lollipop charts depict the CNV frequency of TP53 mutation-related metabolic gene profiles in TP53 mutant **(E)** and wild-type groups **(F)**. **(G, H)** The waterfall plot of tumor somatic mutation in TP53 mutant **(G)** and wild-type groups **(H)**. GO, Gene Ontology; CNV, copy number variation.

First, we used univariate Cox analysis to estimate the survival prognostic value of 92 MRDEGs, and eventually, 24 prognostic genes were determined and regarded as the metabolic genes profile with TP53 mutation (**Figure 2E**). The network diagram depicts the intricate but close correlations and their prognostic significance for BC patients among 24 prognostic genes (**Figure 3A**). Of them, we found 5 prognostic risk factors, as follows: CA9, CHAC1, FUT3, MTHFD1L, and PLCH1. As shown in the heatmap (**Figure 3B**), these 5 risk genes were highly expressed in the mutant TP53 group, and survival analysis indicated that BC cases with elevated expression of these genes were more likely to have a poor survival outcome (**Figure 3C**). Furthermore, Gene Ontology (GO) and KEGG enrichment analyses of the metabolic genes profile were conducted to discover enriched biological processes and activities. We found that they were mainly involved in the regulation of various substances’ metabolism that promoted tumor growth, including amino acids, lipids, and nucleic acids (**Figure 3D**, **Supplementary Table 4**).

We further studied the genomic alterations of 24 key metabolic genes associated with TP53 mutations, thereby deepening our understanding of the metabolic gene profile based on TP53 status. Combined with the copy number information of 1,002 samples and corresponding TP53 status information, 343 patients in the TP53 mutant group and 659 patients in the TP53 wild-type group were used to analyze copy number gain and loss frequencies, respectively. The findings showed that the TP53 mutant group tended to have higher copy number gain or loss frequencies than the TP53 wild-type group (**Figures 3E**, **F**). Next, we also explore 24 metabolic genes mutation between two groups, with detailed TP53 mutation data. As displayed in **Figures 3G**, **H**, the mutation rate of the 24 genes did not differ apparently between the two groups, as well as the overall mutation rate.

### The heterogeneity of TP53 mutation-related metabolic gene profile

**Figure 4:**
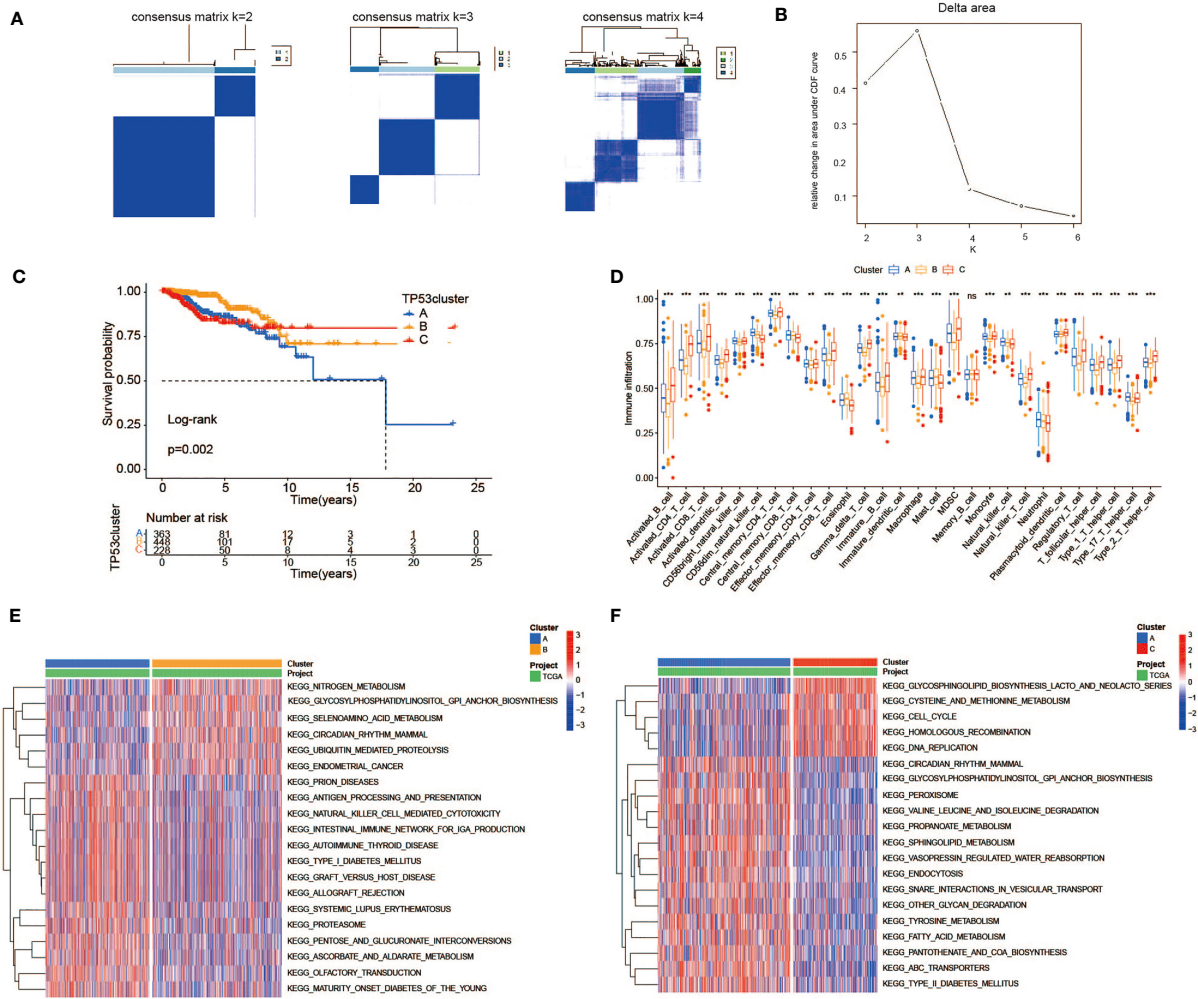
Heterogeneity of TP53 mutation-related metabolic gene profile. **(A)** Consensus clustering matrix of 1,039 TCGA samples for k = 2, k = 3, and k = 4. **(B)** Relative change in area under CDF curve according to various k values. **(C)** Kaplan–Meier survival analysis of three clusters in TCGA samples. **(D)** The abundance of each TME infiltrating cell among three clusters (ns, no significance; **p < 0.01; ***p < 0.001). **(E, F)** Heatmap for GSVA enrichment analysis showing the activation states of biological pathways between Clusters A and B **(E)** and between Clusters A and C **(F)**. TCGA, The Cancer Genome Atlas; CDF, cumulative distribution function; TME, tumor microenvironment; GSVA, gene set variation analysis.

To investigate the metabolic heterogeneity of 92 MRDEGs in BC, 24 prognostic genes were under intensive study. The unsupervised clustering method was utilized to classify patients with qualitatively different metabolic regulation patterns based on the expression of 24 prognostic genes. Considering the principle of high intragroup correlation and low intergroup correlation, we finally determined that the optimal cluster number was three (**Figures 4A**, **B**). We termed these clusters as Clusters A–C: 363 cases in Cluster A, 448 cases in Cluster B, and 228 cases in Cluster C. Furthermore, the Kaplan–Meier survival analysis exhibited striking differences in OS among the three clusters, notably showing a conspicuous survival disadvantage in Cluster A compared with the other two clusters (log-rank p = 0.002, **Figure 4C**). To explore the distinctions in biological processes of the three clusters, we subsequently performed a GSVA enrichment analysis. Obviously, from the perspective of metabolic regulation, Cluster A was mainly enriched in tyrosine metabolism and fatty acid metabolism pathways, Cluster B was closely related to nitrogen metabolism and selenoamino acid metabolism pathways, and Cluster C was intimately associated with pathways that regulate the metabolism of cysteine, methionine, and galactose (**Figures 4E**, **F**, **Supplementary Table 5**). Meanwhile, we sought to further distinguish the three clusters in TME cell infiltration *via* the ssGSEA method. It was worth noting that multiple subpopulations of TILs, presented in **Figure 4D**, were significantly different among the three clusters, revealing immune heterogeneity of TP53 mutation-associated metabolic gene profile in BC patients. Taken together, the discovery of metabolic and immune heterogeneity among the three clusters enabled us to speculate that there may be a complicated pattern of immune metabolic cross-talk regulation in BCs with TP53 mutations.

### Construction of a prognostic risk score model based on metabolic genes (The cancer genome atlas)

**Figure 5:**
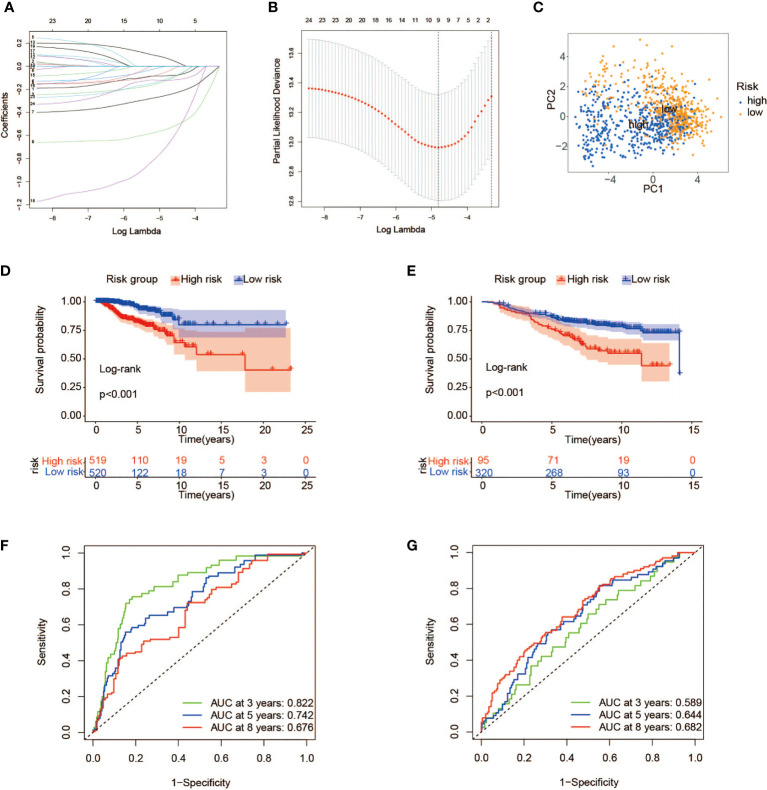
Construction of a prognostic risk model associated with TP53 mutations. **(A, B)** LASSO regression analysis of the 24 metabolic genes associated with TP53 mutations. **(C)** PCA of breast cancer patients with high- and low-risk scores in TCGA. **(D, E)** Kaplan–Meier survival analysis of high- and low-risk groups in TCGA **(D)** and GEO cohorts **(E)**. **(F, G)** ROC curves indicating the sensitivity and specificity of predicting 3-, 5-, and 8-year survival with the TP53 mutation-related signature in TCGA **(F)** and GEO cohorts **(G)**. LASSO, least absolute shrinkage and selection operator; PCA, principal component analysis; TCGA, The Cancer Genome Atlas; GEO, Gene Expression Omnibus; ROC, receiver operating characteristic.

To better quantify the metabolic features and prognosis of each individual, the expression data of 24 metabolic genes associated with TP53 mutations that had been proved above the significance in differentiating BC patients were used to establish a scoring model. Under the application of the LASSO regression model that effectively avoids overfitting conditions, 9 of 24 metabolic genes were retained for the construction of risk scoring formulas with a minimum of λ (**Figures 5A, B**). The specific risk scoring formula is defined as a linear combination of included gene variables weighted by their respective Cox regression coefficients (**Table 1**). Next, we attempted to further probe the clinical implication of this scoring model. In the training cohort, 1,039 samples from TCGA database, with risk scores calculated using the risk scoring formula, were then divided into high- and low-risk groups according to the median score. The risk scatter plot described that BC patients with high-risk scores also have a high risk of death (**Supplementary Figure 2A**). We can see from **Figure 5D** that BC cases at low risk showed a remarkable survival advantage as compared with the high-risk group (log-rank p < 0.001). Furthermore, principal component analysis (PCA) results showed a high degree of differentiation between the high- and low-risk groups (**Figure 5C**). To verify the predictive power of this model, a time-dependent ROC analysis was performed to chart the corresponding ROC curves, and the area under the curve (AUC) was 0.822 for 3-year survival, 0.742 for 5-year survival, and 0.676 for 8-year survival (**Figure 5F**), exhibiting a good predictive accuracy. These findings pointed out that the risk score was expected to be a promising predictor for the prognosis of BC patients.

**Table 1:** Gene variables and their respective coefficients in the risk scoring formula.

Gene	Coefficient
*ABAT*	−0.082288109
*CYP4F8*	−0.124018768
*ELOVL2*	−0.252333184
*ACSM1*	−0.048626551
*GSTM2*	−0.546619727
*CHAC1*	0.070470329
*CD38*	−0.696837951
*PDE6B*	−0.111403019
*UGCG*	−0.1220607

### Validation of the prognostic risk score model

To certify the universal applicability of the scoring model, a testing cohort consisting of 415 BC samples from GSE20685 and GSE20711 in the GEO database has been analyzed. First of all, GEO gene expression data were integrated after removing the batch effect and finally standardized by the “scale” method. A total of 415 BC patients were separated into high-risk (n = 95) and low-risk (n = 320) groups with the same cutoff from the training set (**Supplementary Figure 2B**). As depicted in the Kaplan–Meier curve, a prominent difference in OS did exist between the two groups, and cases at high risk had a worse clinical outcome than those with low-risk scores (**Figure 5E**). Furthermore, ROC curve analysis confirmed the high predictive ability of our model, and the AUC values for 3, 5, and 8 years were 0.589, 0.644, and 0.682, respectively (**Figure 5G**).

### Metabolic characteristics of the high- and low-risk groups

**Figure 6:**
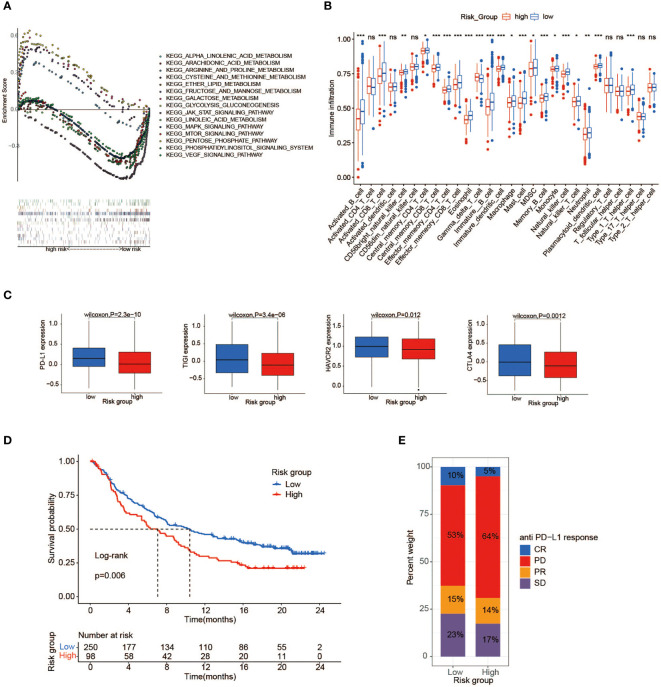
Immune and metabolic differences between high- and low-risk groups. **(A)** GSEA shows a significant enrichment of biological processes in BC patients with low-risk scores compared to those with high-risk scores. **(B)** The abundance of each TME infiltrating cell between high- and low-risk groups (ns, no significance; *p < 0.05; **p < 0.01; ***p < 0.001). **(C)** PD-L1, CTLA4, HAVCR2, and TIGIT gene expression differences between high- and low-risk groups. **(D)** Kaplan–Meier survival analysis of high-risk and low-risk groups in immune cohort IMvigor210. **(E)** The proportion of anti-PD-L1 response in the high- and low-risk groups of the immune cohort IMvigor210. GSEA, gene set enrichment analysis; BC, breast cancer; TME, tumor microenvironment.

To map metabolic alterations between the high- and low-risk groups, we employed a GSEA on 1,039 BC samples from both groups. In the high-risk group, we observed that 17 gene sets representing functions or pathways were significantly upregulated in the KEGG signatures (p < 0.05), among which the main metabolic pathways included glucose metabolism (galactose metabolism, fructose/mannose metabolism, glycolysis/gluconeogenesis, and starch/sucrose metabolism) as well as redox pathways (the pentose phosphate pathway and cysteine/methionine metabolism) (**Figure 6A**, **Supplementary Table 6**). Remarkably, some pathways have been proven to be associated with tumor development and poor prognosis (34–36). In contrast, in the low-risk group, 34 KEGG gene sets were significantly enriched (p < 0.05), with lipid metabolism as the dominant metabolic pathway, typically comprising arachidonic acid metabolism, glycerophospholipid metabolism, alpha-linolenic acid/linoleic acid metabolism, and JAK_STAT signaling pathway (**Figure 6A**). It may reflect that activation of lipid metabolism-related pathways plays a dominant role in tumorigenesis and progression in BC patients with low-risk scores. Finally, there is a bold idea that BC patients in the high-risk group may benefit from therapy targeting glucose MRGs, such as HK3 (37) and PFKP (38). Meanwhile, the novel therapy targeting genes associated with lipid metabolisms, such as HMGCR (39) and FASN (40), may be effective in those at low risk. Before that, further research is indispensable.

### The immune landscape of the high- and low-risk groups

To describe the immune characteristics between low- and high-risk groups, we first calculated the immune infiltrate scores of 28 subpopulations of TILs across TCGA BC samples using the ssGSEA method, and then we performed a differential analysis. Compared with patients in the high-risk group, multiple subpopulations of TILs, such as CD8+ T cells, macrophages, mast cells, natural killer cells, and Type 1 T helper cells (Th1 cells), exhibited enrichment in those with low-risk scores (**Figure 6B**). It was revealed that the low-risk group had an abundance of immune cell infiltration, speculating that patients with low-risk scores may possess more antitumor immunity, such as that mediated by CD8+ T cell. Moreover, we further sought to investigate the correlation between risk scores and important immune checkpoint molecules in TCGA cohorts. As illustrated in **Figure 6C**, the expression of a series of potentially targetable immune checkpoints (PD-L1, CTLA4, HAVCR2, and TIGIT) all tended to be higher in the low-risk group than the high-risk group, implying that low-risk patients may easily benefit from immunotherapy and achieve an improved survival.

**Figure 7:**
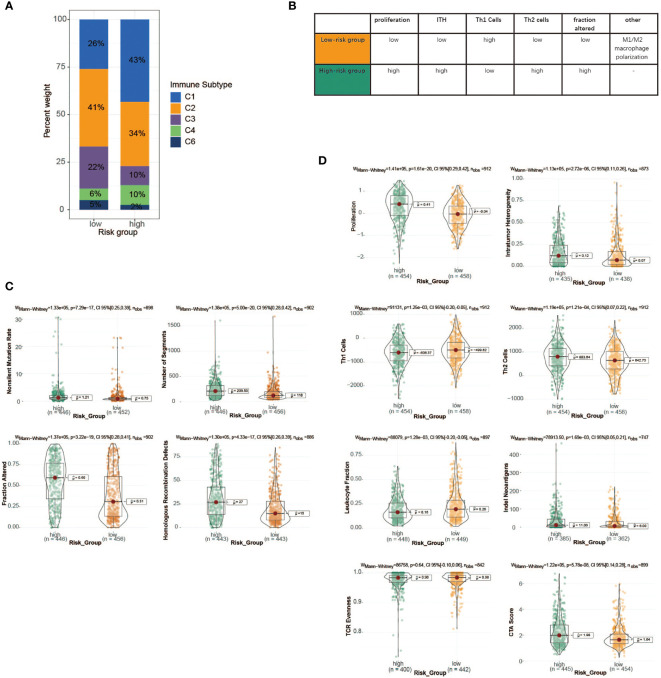
Immune characteristics of the high- and low-risk groups in TCGA cohort. **(A)** The proportion of distinct immune subtypes in the high- and low-risk groups. **(B)** Key characteristics of high- and low-risk groups. **(C)** DNA damage measures of high- and low-risk groups, including non-silent mutation rate, copy number burden scores (number of segments and fraction of genome alterations), and homologous recombination deficiency. **(D)** Values of key immune characteristics between high- and low-risk groups. TCGA, The Cancer Genome Atlas.

To advance this research, we made a meaningful comparison between risk score and 6 immune subtypes (C1–C6) identified by previous researchers based on 30 tumor types (41) (**Supplementary Table 7**). The features of the TME varied substantially across 6 immune subtypes, where C1 was characterized by an increased expression of angiogenic genes, abundant Th2 cell infiltrates, and an elevated proliferation rate, while C2 showed the highest M1/M2 macrophage polarization, a strong CD8 signal, and the greatest T-cell receptor (TCR) diversity. Next, particular immune features of the two groups were then further analyzed (**Figures 7B**–**D**). We observed that Th1 cells and leukocyte fractions were enriched in cases within the low-risk group, which had a high proportion of immune subtype C2, making a major involvement in cell-mediated immunity possible (**Figures 7A**, **B**). In contrast, patients with high-risk scores, consisting mostly of immune subtype C1, showed a high proliferation rate, intratumoral heterogeneity (ITH), and aneuploidy score, which may account for the poor prognosis of cases with high-risk scores.

### The risk score model in the role of anti-PD-1/L1 immunotherapy

We attempted to verify the value of the risk score model to predict the efficacy of anti-PD-1/L1 immunotherapy in other independent cancer cohorts (IMvigor210, GSE78220). The median risk score was selected as the cutoff value to stratify cases into low- and high-risk groups in both cohorts. It was shown that patients with low-risk scores are correlated to a satisfactory prognosis in the IMvigor210 cohort, which was consistent with the previous results (**Figure 6D**). In both cohorts, additional consequences indicated that most cases in the high-risk group displayed discouraging responses to immunotherapy, whereas the low-risk patients showed the opposite (**Figure 6E**, **Supplementary Figure 3B**). Although the exploration and analysis of the relationship between risk score and clinical efficacy did not show a marked difference, they still displayed a trend of poor responses in high-risk cases (**Supplementary Figures 3C**, **E**). Intriguingly, in comparison with the high-risk group, the elevated expression of the PD-L1 gene as a pivotal target for immunotherapy was observed in the low-risk group, possibly contributing to favorable responses to anti-PD-1/L1 therapy (**Supplementary Figures 3D**, **F**).

### Independence evaluation of risk score model

**Figure 8:**
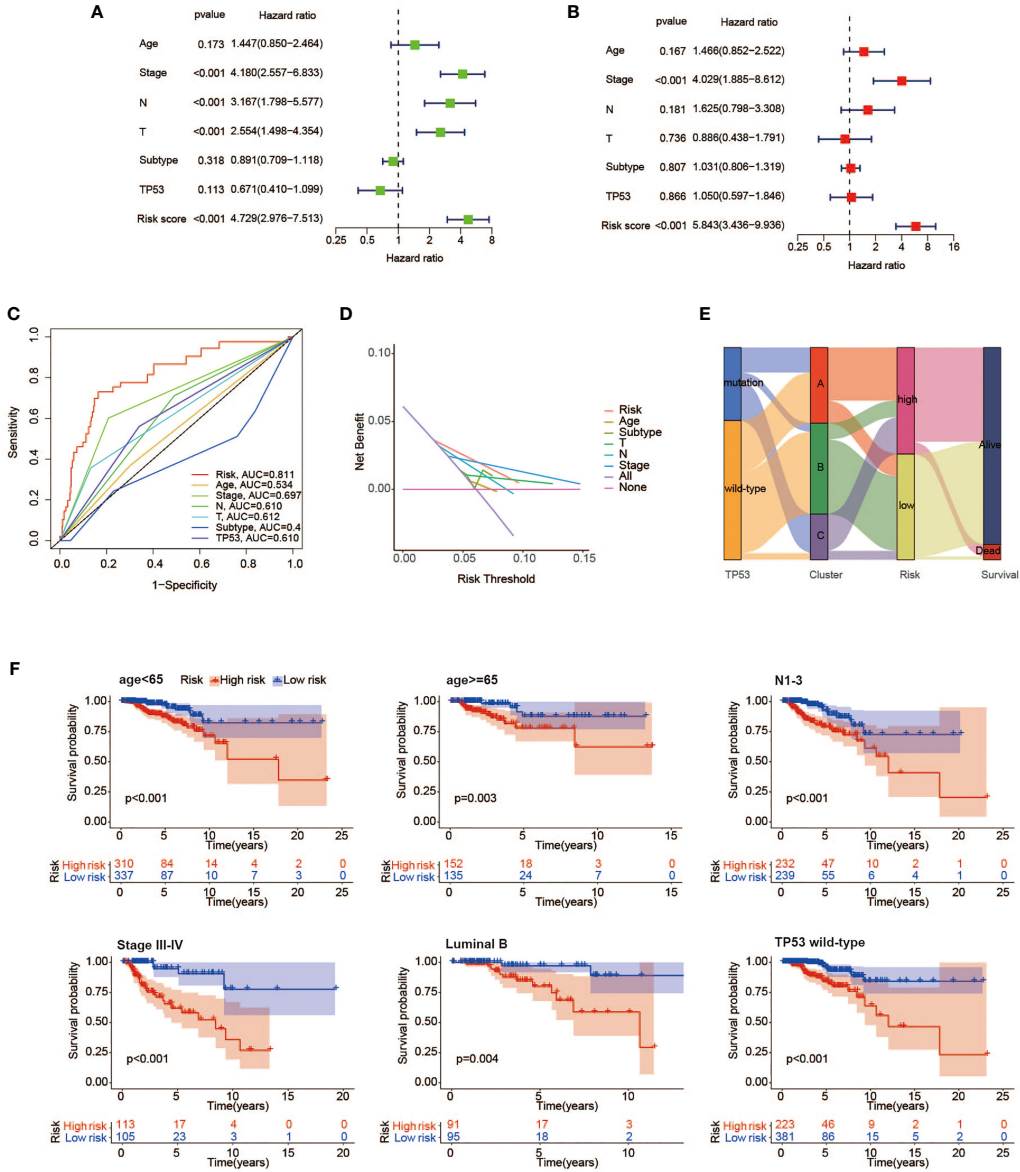
Independence evaluation of the prognostic risk model. Univariate **(A)** and multivariate **(B)** analyses of traditional clinical features and the prognostic risk model. **(C)** ROC curves indicating the sensitivity and specificity of predicting 3-year survival with traditional clinical features and the prognostic risk model in TCGA cohort. **(D)** Decision curves for traditional clinical features and the prognostic risk model to predict 3-year survival probability. **(E)** Alluvial diagram showing the changes of TP53 condition, molecular clusters, risk group, and survival status. **(F)** Kaplan–Meier survival analysis of high- and low-risk groups in the <65 years, Luminal B, N1–3, stage III–IV, TP53 mutant, and wild-type subgroups. ROC, receiver operating characteristic; TCGA, The Cancer Genome Atlas.

A total of 934 BC samples with detailed clinical features were obtained from TCGA and summarized in **Table 2**. A univariate Cox regression model was performed to seek the prognostic connection between OS time for BC cases and several special characteristics, including age, clinical stage, T classification, N classification, molecular subtype, TP53 condition, and risk score. Among them, only 4 significant factors with a p-value <0.001 were closely related to OS and subsequently incorporated into the multivariate Cox analysis (**Figures 8A**, **B**). Collectively, the result in **Figures 8C–E** underlined the stronger role of risk score based on our model as an independent prognostic factor for BC patients.

**Table 2:** Baseline characteristics of breast cancer cases in TCGA cohort.

Characteristics	TCGA
Patients (n)	934
Mean follow-up time (months, range)	27.0 (0, 282.9)
Age (years)	
**<**65	647
≥65	287
Stage	
I–II	716
III–IV	218
AJCC pathologic T	
T1–2	797
T3–4	137
AJCC pathologic N	
N0	463
N1–3	471
AJCC pathologic M	
M0	794
M1	15
Mx	124
Risk group	
Low	472
High	462
TP53	
Wild type	604
Mutant	330
Subtype	
BRCA_Normal	34
BRCA_LumA	477
BRCA_LumB	186
BRCA_Her2	72
BRCA_Basal	165

TCGA, The Cancer Genome Atlas; AJCC, American Joint Committee on Cancer.

To further evaluate the predictive sensitivity of the risk score in distinct stratified subgroups, the differences in OS between the high- and low-risk subgroups were compared by using the Kaplan–Meier survival analysis in TCGA cohort. In most subgroups, it was well demonstrated that BC cases with high-risk scores were linked to worse prognosis, especially in the <65 years, Luminal A, Luminal B, T3–4, N1–3, stage III–IV, and TP53 wild-type subgroups (**Figure 8F**).

The following analyses were also presented for the correlation between risk scores and various clinicopathological features (**Supplementary Figure 4**). As a result, patients in the TP53 mutant subgroup were indicative of a higher risk score in comparison to those without mutated TP53, which reflected poorer survival outcomes as well. In terms of molecular subtype, patients with basal-like subtype and her2-enriched subtype were close related to high-risk scores. Moreover, we found that several known risk factors, such as larger tumor size, lymph node metastasis, and advanced clinical stage, were accompanied by superior risk scores.

## Discussion

Considering the high heterogeneity of BC, clinicians are confronted with great challenges in the improvement of the survival rate for BC patients who exhibit poor responses to treatment (11, 42). With the advances in medicine, multiple novel approaches have emerged as indispensable tools in patient classification, disease status monitoring, and personalized treatment regimens, including molecular biomarkers, prognosis, and diagnostic gene signatures (43, 44). Therefore, it is necessary to explore the promising genetic signatures to better predict the prognosis of BC patients and assist in making individualized treatment options.

Currently, many researchers have proved that the status of *TP53* gene is closely related to the prognosis of BC (45, 46), but it could not improve the prognostic accuracy in the absence of comprehensive bioinformatics and clinicopathological factors analysis. In this study, we are the first to identify a metabolic gene profile associated with TP53 mutations using a large cohort of TCGA BC patients and, further, reveal its underlying immunological and metabolic heterogeneity. Furthermore, a powerful TP53 mutation-related prognostic model was figured out *via* the LASSO regression methods and validated in two GEO datasets. Meanwhile, through various bioinformatics methods, we summarized that patients with different risk scores varied in the immune microenvironment, metabolic activities, and responses to immunotherapy, providing a guide for targeted metabolic therapy of BC. This is also a prognostic model that goes beyond traditional clinical features and a single gene to accurately identify those patients with poor survival and better guide clinical therapy.

Mutant TP53 has attracted much attention in tumorigenesis and development since its first description in 1989 (47). Clinically, patients with mutant TP53 have been associated with a discouraging prognosis in various cancers, but the results remain controversial (48). In a study of 859 BC patients, the researchers determined that patients with mutant TP53 had worse BC-specific and all-cause mortality than those with wild-type TP53, which is consistent with other studies (17–19). In our study, we finally illustrated no statistically significant difference in survival between the TP53 mutant and wild-type groups from TCGA cohort of 1,003 BC patients. Currently, many researchers have proved that the status of *TP53* gene is closely related to the prognosis of BC (45, 46), but it could not improve prognostic accuracy in the absence of comprehensive bioinformatics and clinicopathological factors analysis. In this study, we are the first to identify a metabolic gene profile associated with TP53 mutations using a large cohort of TCGA BC patients and, further, reveal its underlying immunological and metabolic heterogeneity. Furthermore, a powerful TP53 mutation-related prognostic model was figured out *via* the LASSO regression methods and validated in two GEO datasets. Meanwhile, through various bioinformatics methods, we summarized that patients with different risk scores varied in the immune microenvironment, metabolic activities, and responses to immunotherapy, providing a guide for targeted metabolic therapy of BC. This is also a prognostic model that goes beyond traditional clinical features and a single gene to accurately identify those patients with poor survival and better guide clinical therapy. In this regard, it may be caused by differences in clinical characteristics or treatment options that affect prognostic determinations. Of note, a large study of the METABRIC dataset (n = 1,979) elucidated the clear relationship between TP53 mutation status and survival in different therapy regimens. Consequently, in patients treated with hormone replacement therapy (HRT) only, those without TP53 mutation had a survival advantage. Conversely, the TP53 mutant patients obtained a superior rate of pathologic complete response (pCR) when chemotherapy was only administrated (45, 49, 50). In brief, the profound impact of TP53 on the prognosis of BC is indisputable.

Accumulating evidence suggests that *TP53* gene is essential for cancer initiation and progression by reprogramming cancer cell metabolism in addition to p53-mediated classical regulatory mechanisms (51). In our research, we identified 92 metabolic genes related to TP53 mutation, and results of KEGG analysis showed that mutated TP53 principally participated in glycolysis/gluconeogenesis, tryptophan metabolism, glutathione metabolism, glycosphingolipid biosynthesis, and purine metabolism, which may promote tumor progression *via* meeting increased demands for energy, biomass, and nutrients. Some mechanistic research revealing detailed mutant p53-mediated metabolic regulatory pathways has been reported. A study in 2013 demonstrated that mutated p53 protein assisted GLUT1 transport to the plasma membrane and enhanced glycolysis and tumorigenesis *via* a RhoA/ROCK/GLUT1 signaling pathway (52). Additionally, it has been revealed that mutated p53 tended to bind with the AMP-activated protein kinase (AMPK) α subunit and then restrained its activation, resulting in increased lipid production and tumor growth in the head and neck cancer cells (53). Although there are more and more in-depth mechanistic studies mentioned above, which provide a theoretical basis for targeted metabolic therapy, metabolic flexibility that enables cancer cells to adapt to the microenvironmental perturbations and metabolic heterogeneity are regarded as critical barriers to targeting cancer metabolic profiles (54, 55). Our subsequent research results referring to TP53 mutation-related metabolic gene profiles showed that enrichment of metabolic pathways varied in three clusters, which embodied metabolic heterogeneity. Cluster A was enriched in fatty acid metabolism and tyrosine metabolism, but it had the worst prognosis among the three clusters. This can be explained by some standpoints in several studies that aberrantly activated fatty acid metabolism, including synthesis, lengthening, and desaturation can facilitate tumor proliferation (56, 57). However, our findings indicated that Cluster A was expected to benefit from targeted therapy of fatty acid metabolism.

Using TCGA cohort data as a training set, we constructed a prognostic risk scoring model based on 9 metabolic genes associated with TP53 to quantitatively score the risk of an individual BC patient, so as to improve individualized cancer treatment and monitoring. GEO data of 415 BC cases were used as a validation set, which well verified the good accuracy of our model. We then used the median risk score to classify patients into the high- and low-risk groups. It has been revealed that various metabolic mechanisms have an intricate relationship with the behavior of immune cells and antitumor immune response and are involved in the process of tumor genesis and development (58). Therefore, we sought to uncover the distinct immunological landscape and heterogeneity of metabolic profiles between the high- and low-risk groups. First, our results showed that compared with the high-risk prognosis group, higher abundance of most immune cells, whether immunosuppressive cells (such as Tregs, tumor-associated macrophages (TAMs), and myeloid-derived suppressor cells (MDSCs)) or immune effector cells, was observed in the low-risk prognosis group, highlighting the existence of a complex internal immune microenvironment. Further studies indicated that IFN-γ signaling was dominant in the low-risk group, with high M1/M2 macrophage polarization and strong CD8 signaling. Meanwhile, CD8+ T cell was accepted as a crucial determinant of favorable clinical prognosis in patients with BC (59). Of interest, our study found that cases in the low-risk prognosis group had elevated expression of PD-L1, CTLA4, HAVCR2, and TIGIT and were more sensitive to anti-PD1 treatment than those with high-risk scores. Taken together, these results suggest that good outcomes in the low-risk group may be associated with immune effector cell-dominated antitumor immunity and those patients may benefit from immunotherapy. For metabolic activities, significant enrichment of lipid-related metabolism was found in the low-risk group, with compelling evidence from other literature showing abnormally activated lipid metabolism in BC. Previous studies have demonstrated that JAK/STAT signaling pathway is closely related to BC stem cells and chemotherapy resistance, mainly by inhibiting fatty acid β-oxidation (FAO) (60). Moreover, arachidonic acid is an important component of phospholipids in cell membranes, and its metabolism is critical for the migration of BC cells induced by oleic acid (61). At present, metabolic-targeting therapy strategies have been put into practice, with many drugs targeting metabolic enzymes in clinical trials, so our results provide a precise direction on lipid metabolism for BC. It is interesting to note that other literature has reported that activated T cells can upregulate lipid synthesis and cholesterol uptake to reprogram lipid metabolism (61, 62). Thus, we hypothesized that the complicated pattern of immunometabolic intermodulation played an irreplaceable role in BC survival.

According to the Cox proportional hazards model, univariate and multivariate analyses were utilized to identify clinical stage and risk sore as independent prognostic factors for BC survivors. This result also indicates that the excellent prognostic ability of our risk scoring model for BC is comparable to the clinical stage and even better than age and molecular subtypes. In terms of predicting the 3-year survival of BC patients, ROC curve results intuitively suggested that our model had higher accuracy than traditional clinical features. Subsequently, we performed decision curve analysis (DCA) to show the superior clinical utility of the risk model, which was supported by our discovery that our model can be widely applied to different clinical subgroups.

Certainly, there are some shortcomings that arise in the present study. First, the data of our study cohort are all obtained from public databases, which may be incapable of representing the entire population of BC patients due to large heterogeneity. Another limitation is that our study could not thoroughly explore the relationship between the TP53 condition and different treatment options for BC survivors because of incomplete data on treatment regimens. Finally, our nomogram and risk scoring system are limited by the retrospective nature of data collection, so it is necessary to develop further prospective studies to validate our findings.

In conclusion, we identified 24 metabolic genes associated with TP53 mutations and defined them as metabolic gene profiles, which are conducive to a deeper understanding of metabolic pathway changes caused by TP53 mutations and provide therapeutic targets for targeted metabolic pathways for BC patients with TP3 mutations. Second, five risk metabolism genes (*CA9*, *CHAC1*, *FUT3*, *MTHFD1L*, and *PLCH1*) were found to confer potential for targeted therapy. In addition, the risk score model based on TP53-related metabolic genes was constructed and verified for the first time, providing a new prediction method for the prognosis of BC and contributing to the clinical decision making and dynamic monitoring of individuals.

## Data Availability Statement

The datasets analyzed for this study can be found in the online repositories. The names of the repository/repositories and accession number(s) can be found in the article.

## Author Contributions

MJ, XW (2nd Author), and SB contributed to the conception and design of the study. MJ and XW (2nd Author) obtained the online datasets. MJ and SB performed the statistical analysis. MJ wrote the first draft of the manuscript. SB, FQ, QL, XW (4th Author), XH, WL, JT, and YY contributed to the revision of the manuscript. All authors contributed to the article and approved the submitted version.

## Funding

This work was financially supported by the National Key Research and Development Program of China (ZDZX2017ZL-01), High-level Innovation Team of Nanjing Medical University (JX102GSP201727), Wu Jieping Foundation (320.6750.17006), Key Medical Talents (ZDRCA2016023), 333 Project of Jiangsu Province (BRA2017534 and BRA2015470), The Collaborative Innovation Center for Tumor Individualization Focuses on Open Topics (JX21817902/008), and Project of China Key Research and Development Program Precision Medicine Research (2016YFC0905901).

## Conflict of Interest

The authors declare that the research was conducted in the absence of any commercial or financial relationships that could be construed as a potential conflict of interest.

The reviewer JL declared a shared affiliation with the authors MJ, SB, XW, FQ, QL, XH, WL, JT and YN, to the handling editor at time of review.

## Publisher’s Note

All claims expressed in this article are solely those of the authors and do not necessarily represent those of their affiliated organizations, or those of the publisher, the editors and the reviewers. Any product that may be evaluated in this article, or claim that may be made by its manufacturer, is not guaranteed or endorsed by the publisher.
