# Supplementary material for: Immunometabolism characteristics and a potential prognostic risk model associated with TP53 mutations in breast cancer
Source: Front Immunol. 2022 Jul 22;13:946468. doi: 10.3389/fimmu.2022.946468 (PMC9353309; doi:10.3389/fimmu.2022.946468)
Supplement: Supplementary file 1 [file DataSheet_1.zip › Suppl_Image/Suppl_Image.docx]

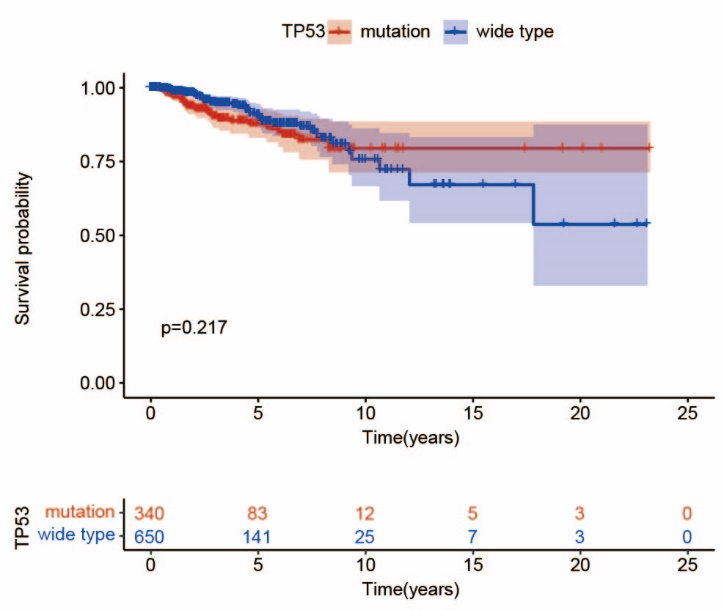


**Supplementary Figure 1.** Survival analysis of TP53 mutant and wide type groups in TCGA samples.


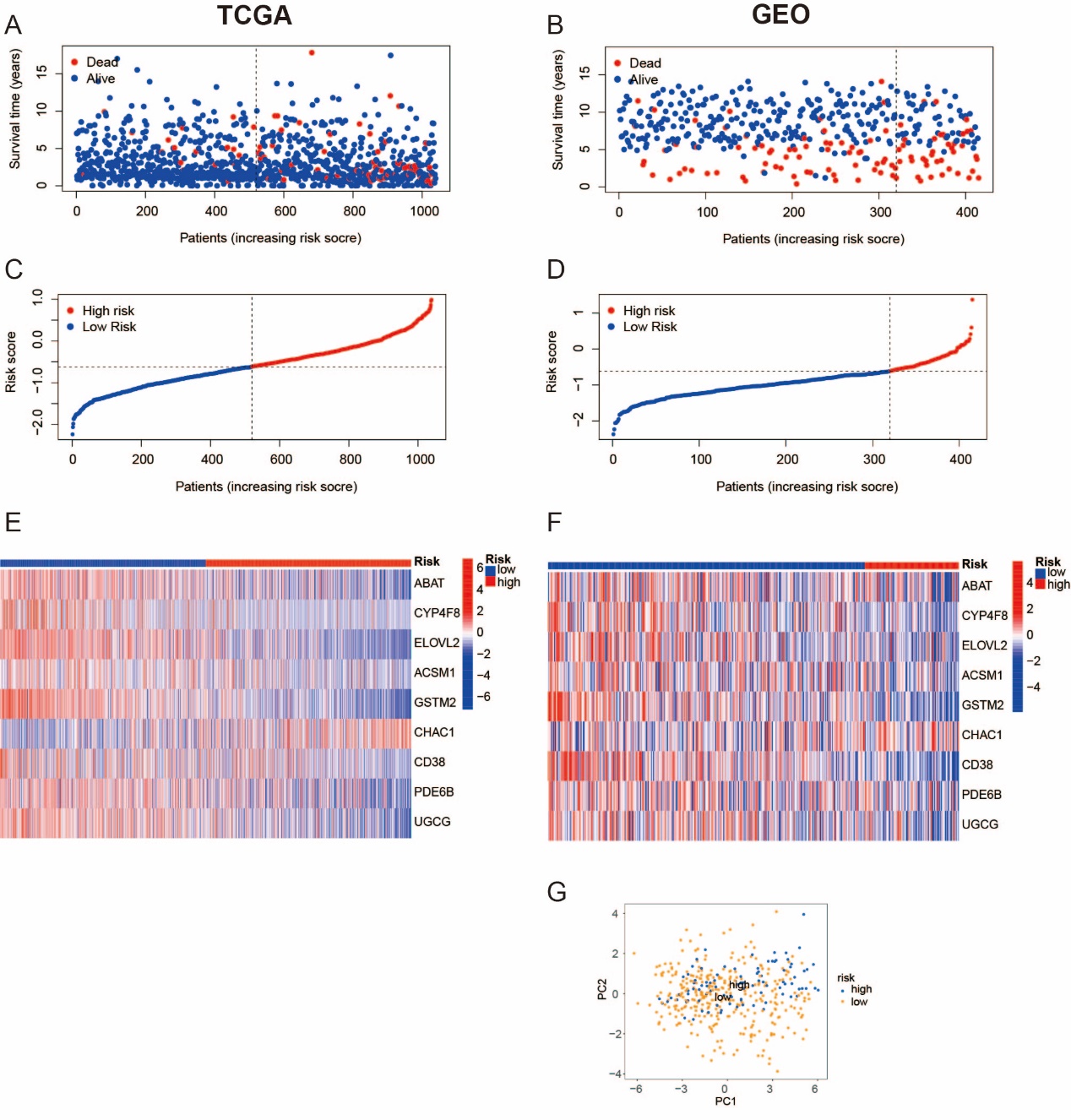


**Supplementary Figure 2.** Construction and validation of the prognostic risk model in the TCGA and GEO cohort. **(A, B)** The risk score distribution of BC patients in the TCGA**(A)** and GEO cohort**(B)**. **(C, D)** Survival status and duration of BC patients in the TCGA**(C)** and GEO cohort**(D)**. **(E, F)** Heatmap of the 9 metabolic genes expression included in the prognostic risk model from the TCGA**(E)** and GEO cohort**(F)**. **(G)**PCA analysis of breast cancer patients with high-risk scores and low risk scores in GEO.


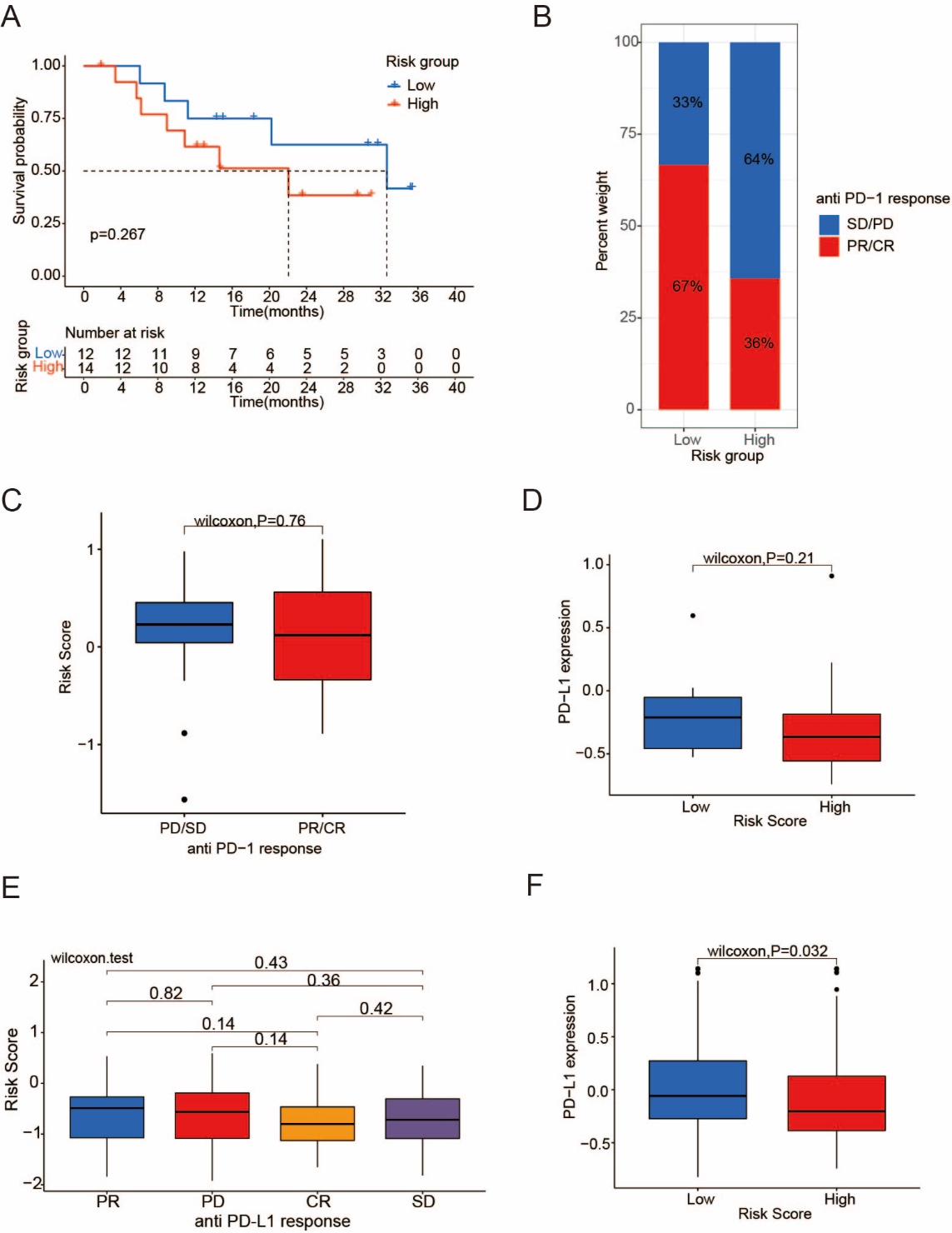


**Supplementary Figure 3.** Correlation between risk score and response to immunotherapy in immune cohorts. **(A)** Kaplan-Meier survival analysis of high-risk and low-risk groups in immune cohort GSE78220. **(B)**Proportion of anti PD-1 response in the high risk and low risk groups of the immune cohort GSE78220. **(C, E)** correlation between risk score and anti-PD-1/L1 response in immune cohort GSE78220**(C)** and IMvigor210**(E)**. **(D, F)** PD-1/L1 gene expression between high risk and low risk groups in immune cohort GSE78220**(D)** and IMvigor210**(F)**.


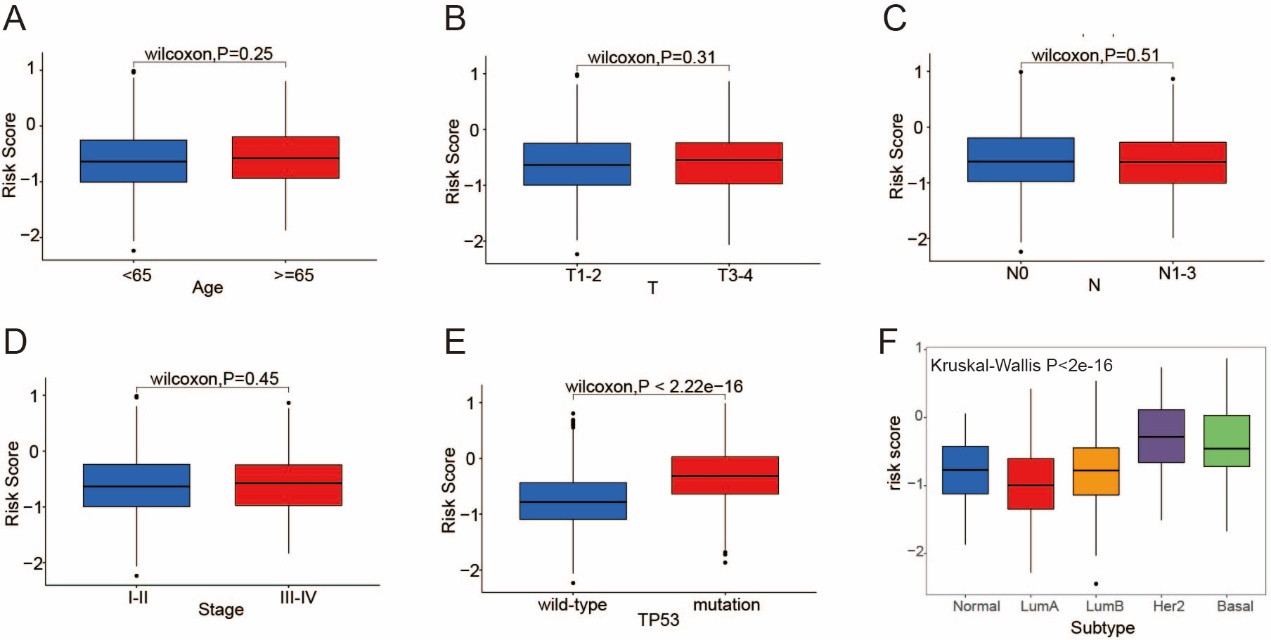


**Supplementary Figure 4.** Correlation between risk score and various clinicopathologic features in TCGA cohort. **(A-F)** Correlation between risk score and various clinicopathologic features, including Age**(A)**, T**(B)**, N**(C)**, Stage**(D)**, TP53 condition**(E)**, Subtype**(F)**.
