# Supplementary figures and images for: Immunometabolism characteristics and a potential prognostic risk model associated with TP53 mutations in breast cancer
Source: Front Immunol. 2022 Jul 22;13:946468. doi: 10.3389/fimmu.2022.946468 (PMC9353309; doi:10.3389/fimmu.2022.946468)

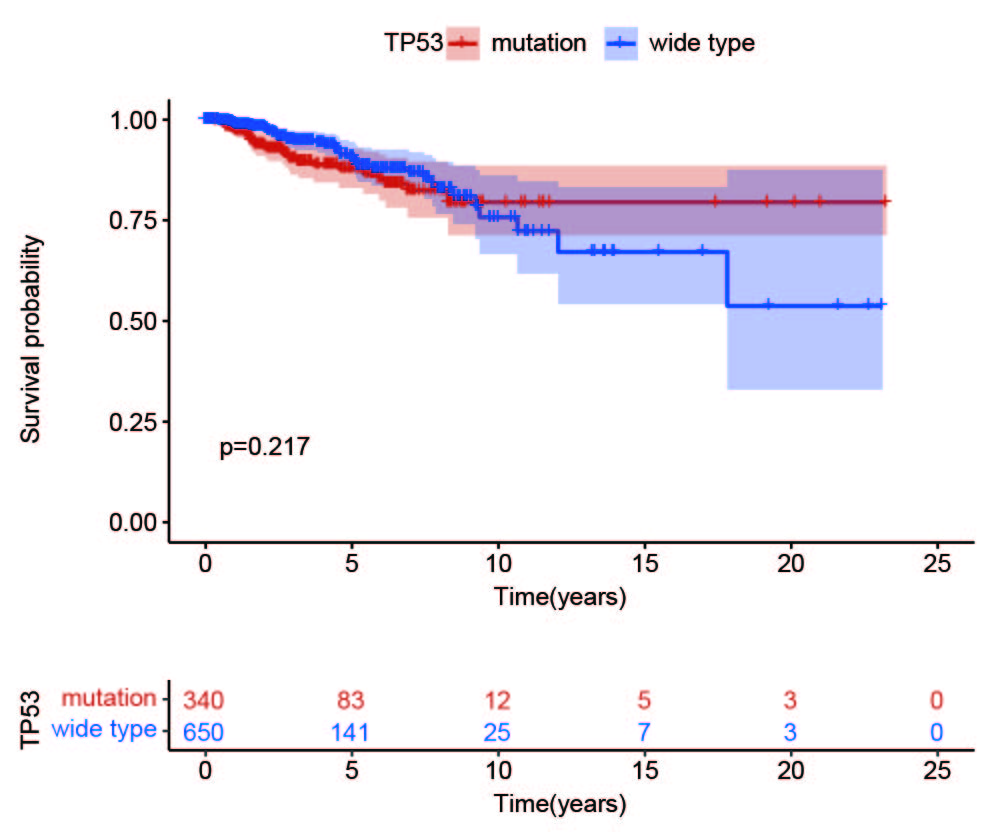

Supplement: Supplementary file 1 [file DataSheet_1.zip › Suppl_Image/Supplementary Figure 1.jpg]

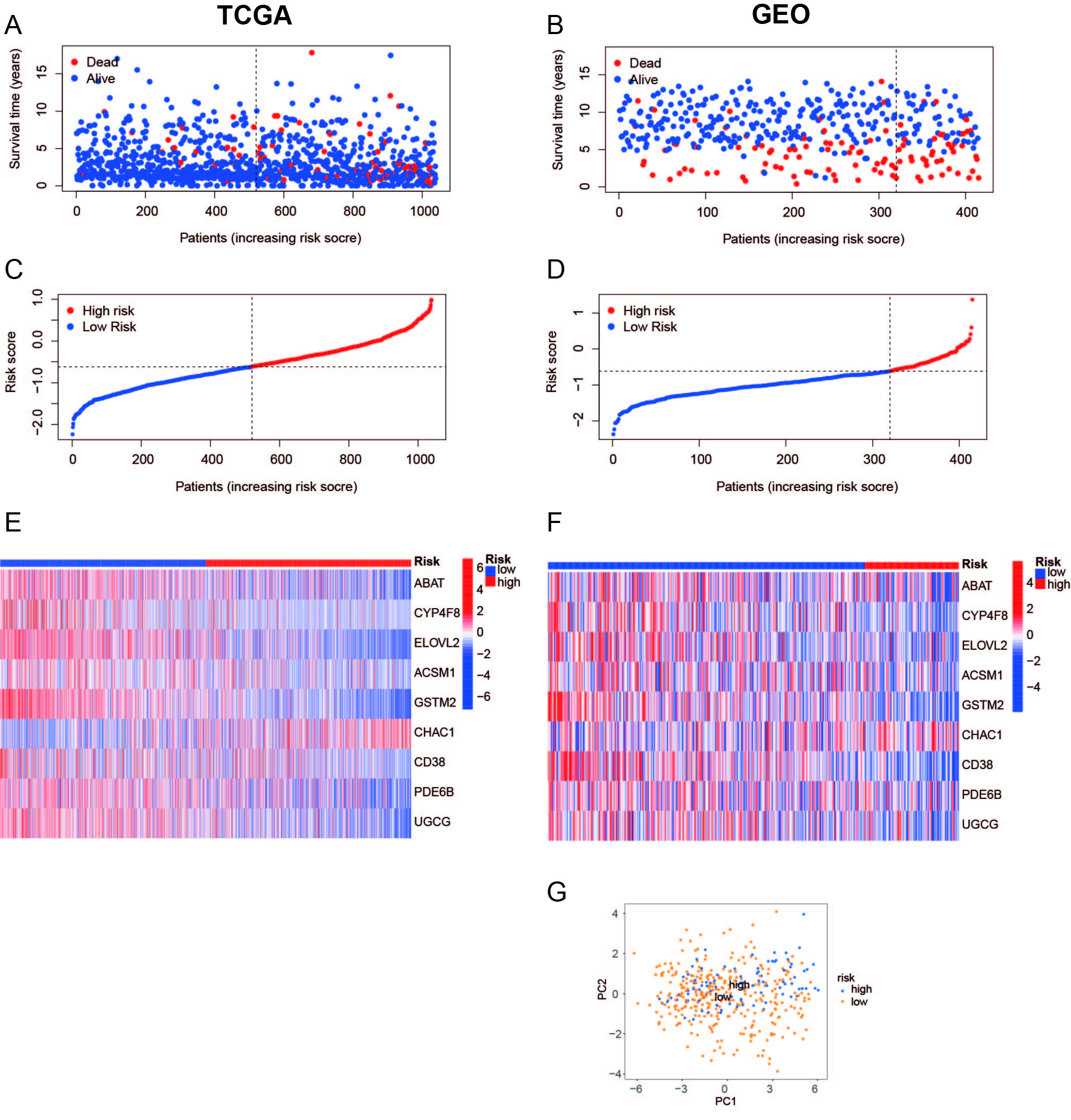

Supplement: Supplementary file 1 [file DataSheet_1.zip › Suppl_Image/Supplementary Figure 2.jpg]

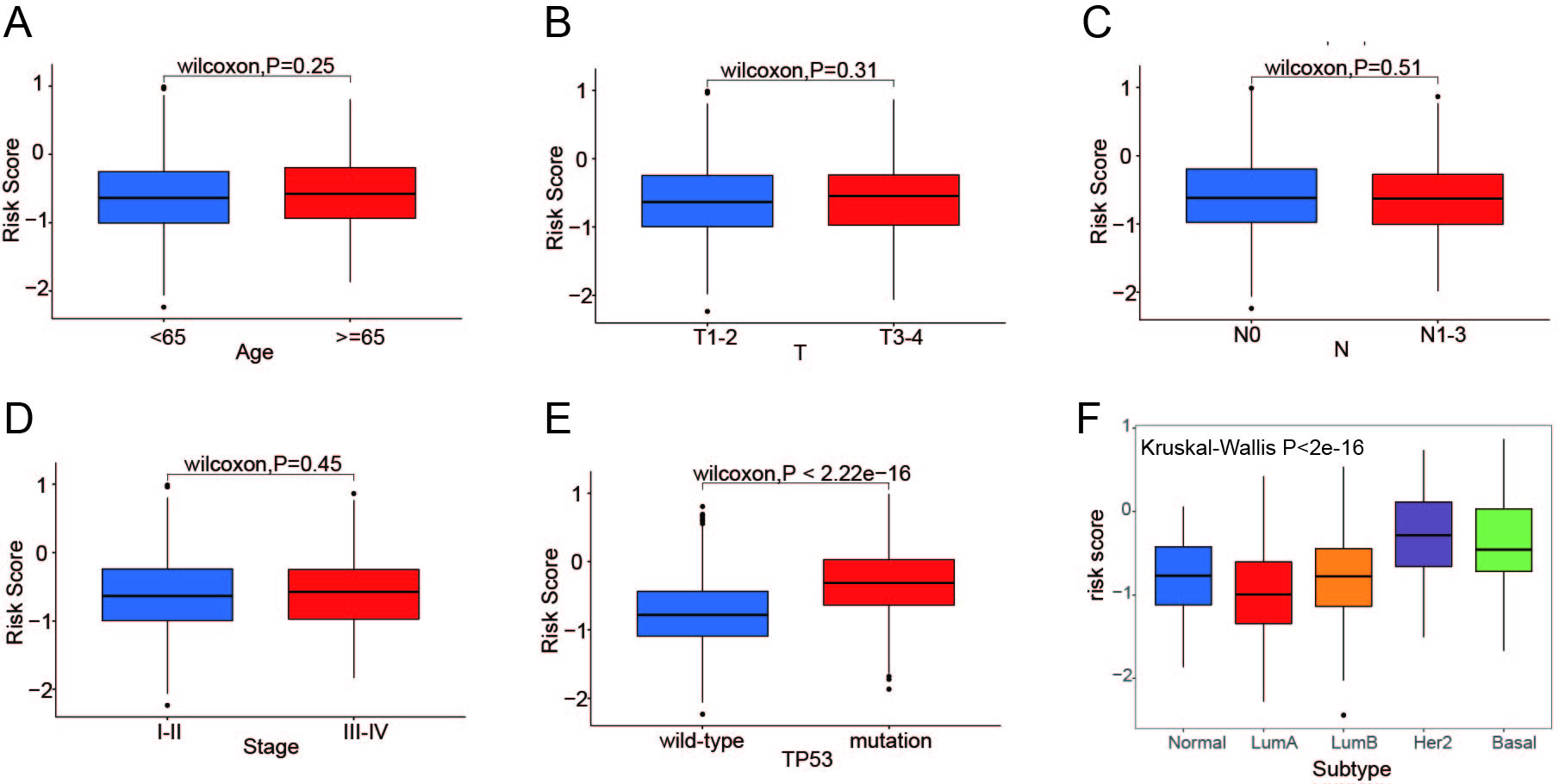

Supplement: Supplementary file 1 [file DataSheet_1.zip › Suppl_Image/Supplementary Figure 4.jpg]

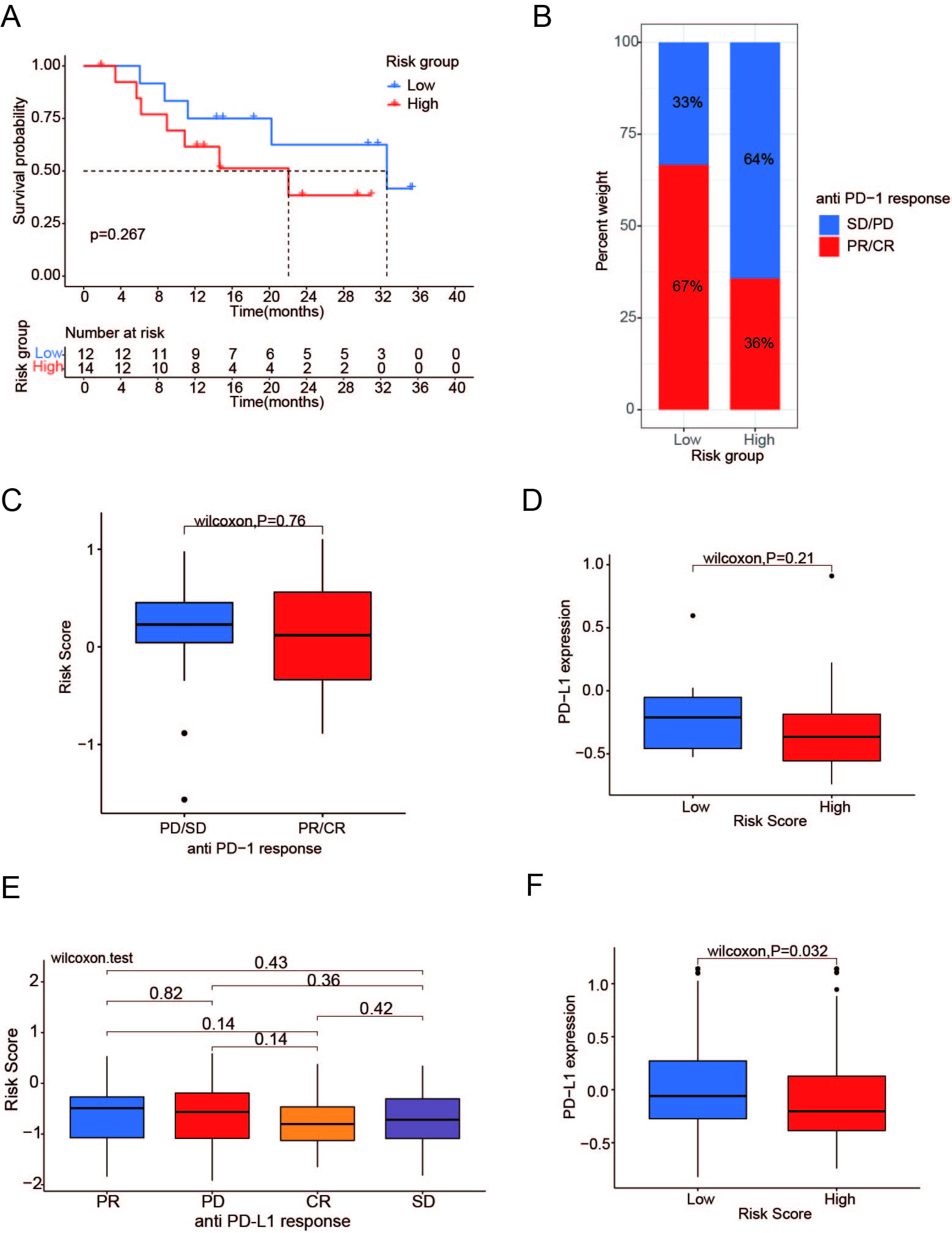

Supplement: Supplementary file 1 [file DataSheet_1.zip › Suppl_Image/Supplemntary Figure 3.jpg]
